# Prevention and treatment of osteoporosis with natural products: Regulatory mechanism based on cell ferroptosis

**DOI:** 10.1186/s13018-023-04448-3

**Published:** 2023-12-11

**Authors:** Yunshang Yang, Yifan Jiang, Daoyi Qian, Zhirong Wang, Long Xiao

**Affiliations:** 1 Translational Medical Innovation Center, The Affiliated Zhangjiagang TCM Hospital of Yangzhou University, Zhangjiagang, 215600 Jiangsu China; 2 Department of Orthopedics, The Affiliated Zhangjiagang TCM Hospital of Yangzhou University, Zhangjiagang, 215600 Jiangsu China

**Keywords:** Ferroptosis, Natural products, Osteoporosis, Osteoblast, Osteoclast

## Abstract

**Context:**

With the development of society, the number of patients with osteoporosis is increasing. The prevention and control of osteoporosis has become a serious and urgent issue. With the continuous progress of biomedical research, ferroptosis has attracted increased attention. However, the pathophysiology and mechanisms of ferroptosis and osteoporosis still need further study. Natural products are widely used in East Asian countries for osteoporosis prevention and treatment.

**Objective:**

In this paper, we will discuss the basic mechanisms of ferroptosis, the relationship between ferroptosis and osteoclasts and osteoblasts, and in vitro and in vivo studies of natural products to prevent osteoporosis by interfering with ferroptosis.

**Methods:**

This article takes ferroptosis, natural products, osteoporosis, osteoblasts and osteoclast as key words. Retrieve literature from 2012 to 2023 indexed in databases such as PubMed Central, PubMed, Web of Science, Scopus and ISI.

**Results:**

Ferroptosis has many regulatory mechanisms, including the system XC -/GSH/GPX4, p62/Keap1/Nrf2, FSP1/NAD (P) H/CoQ10, P53/SAT1/ALOX15 axes etc. Interestingly, we found that natural products, such as Artemisinin, Biochanin A and Quercetin, can play a role in treating osteoporosis by promoting ferroptosis of osteoclast and inhibiting ferroptosis of osteoblasts.

**Conclusions:**

Natural products have great potential to regulate OBs and OCs by mediating ferroptosis to prevent and treat osteoporosis, and it is worthwhile to explore and discover more natural products that can prevent and treat osteoporosis.

## Introduction

Osteoporosis is a disease that affects the skeletal system throughout the body and is mainly characterized by increased brittleness of the bones and decreased bone mass, which predisposes individuals to fractures [1]. The incidence of osteoporosis is increasing every year with the development of society and the decreasing birth rate. According to one study, approximately 8.9 million people worldwide experience fractures every year. [2], and the risk of fracture increases from 60 to 82% per 10,000 patients per year [3]. The main risk factor for such fractures is osteoporosis, with brittle fractures being more common [4]. Bone health depends on the balance between bone formation and bone resorption. However, when this balance is disturbed, osteoporosis can occur. Osteoclasts (OCs) are the main players in bone resorption. OCs are responsible for bone resorption, which can be divided into the following pathways: bone adsorption, cytoskeletal reorganization and vesicular transport [5]. Bone formation is dominated by osteoblasts (OBs), which are capable of mediating bone formation through runt-related transcription factor 2 (Runx2) [6]. Runx2 is not only a key regulator of OB maturation but also regulates OB extracellular matrix components, such as osteocalcin (OCN), osteopontin (OPN) and bone salivary protein (BSP) [7]. The expression and transcription of these factors, in turn, promote OB maturation [8]. In addition, there are also some factors that directly regulate osteoporosis, such as regulating BMD by mediating Vitamin D Receptor [9]. Alendronate [10] and Denosumab [11, 12] are the preferred drugs for treating osteoporosis in clinical practice, and experiments have also confirmed that they have achieved good clinical efficacy. It is worth mentioning that when observing the efficacy of medication in treating osteoporosis, bone turnover biochemical markers are essential [13, 14]. However, the above-mentioned drug treatment mechanisms are relatively single, and in order to enrich the treatment methods for osteoporosis, it is particularly important to find new drugs and new mechanisms for preventing and treating osteoporosis.

Ferroptosis is a form of programmed cell death characterized by iron-mediated accumulation of lipid peroxidation leading to increased density and contraction of mitochondrial membranes [15]. Ferroptosis was officially named by Scott J Dixon and colleagues in 2012 after the discovery that erastin triggered a unique iron-dependent form of nonapoptotic cell death in oncogenic RAS-selective models [16]. The morphological characteristics of ferroptosis are an increase in mitochondrial membrane density and a decrease in mitochondrial volume, as well as a disruption in outer mitochondrial membrane integrity, resulting in the dissolution and disappearance of mitochondrial cristae [17].

Ferroptosis is widely used in the regulation of major systemic diseases such as cancer [18], liver disease [19], Alzheimer's disease [20], and cardiovascular disease [21]. In recent years, researchers have also focused on ferroptosis-mediated regulation of osteoporosis [22–24]. Studies have confirmed that ferroptosis regulates osteoporosis by inhibiting OC-mediated bone resorption and promoting bone formation by OBs [25]. Alireza V enhanced the bone formation capacity and cellular activity in OBs by using the iron-lowering inhibitor ferrostatin-1 in cancer cells, as determined by examining cell differentiation, alizarin red staining and RUNX2 gene expression [26]. Several studies have demonstrated that melatonin can reduce steroid-induced osteoporosis and diabetic osteoporosis by inhibiting OCs and promoting the ferritin pathway in OBs [27–29]. Ferroptosis-mediated regulation of osteoporosis via herbal medicine and herbal compounds has also received increasing attention from researchers in East Asian countries.

East Asian countries, especially in China, have rich experience in the use of natural products. They have the advantages of low price, multi-target synergy and broad research prospects. Based on these, this paper summarizes the mechanisms and regulatory pathways of ferroptosis, and the regulation of osteoblasts and osteoclasts. At the same time, the in vivo and in vitro studies on the prevention and treatment of osteoporosis by some natural products through ferroptosis were discussed. It is hoped that this review can provide the necessary theoretical basis for the prevention and treatment of osteoporosis by natural products through regulating ferroptosis.

## Mechanisms and regulation of ferroptosis

### Iron metabolism associated with Ferroptosis in vivo

Iron is one of the essential trace elements in the human body and plays an important role in cell proliferation and function [30]. The theory that iron overload due to abnormal iron metabolism is the main feature of ferroptosis has been recognized by researchers [15, 16, 31]. In the human body, iron is widely present and mainly in the form of ferrous ions (Fe^2+^) and ferric ions (Fe^3+^). Circulating iron binds to transferrin receptor 1 (TFR1) on the cell membrane, and subsequently, Fe^3+^ is reduced to Fe^2+^ by the six-transmembrane epithelial antigen of prostate 3 (STEAP3) [32, 33]. Divalent metal transporter protein 1 (DMT1) releases Fe^2+^ into a labile iron pool (LIP) in the cytoplasm [34]. It is important to recall that the LIP enables the active uptake of free iron in the cytoplasm as well as the recycling of iron from ferritin and mitochondria. There is a large LIP in lysosomes [35]. Therefore, the main organelle associated with ferroptosis is also one of the targets of disease treatment [36]. Immediately afterward, ferritin 1 (FPN1) transports excess Fe^2+^ outside the cell and stores it in ferritin heavy chain 1 (FTH1) and ferritin light chain 1 (FTI1) [37, 38].

**Fig. 1:**
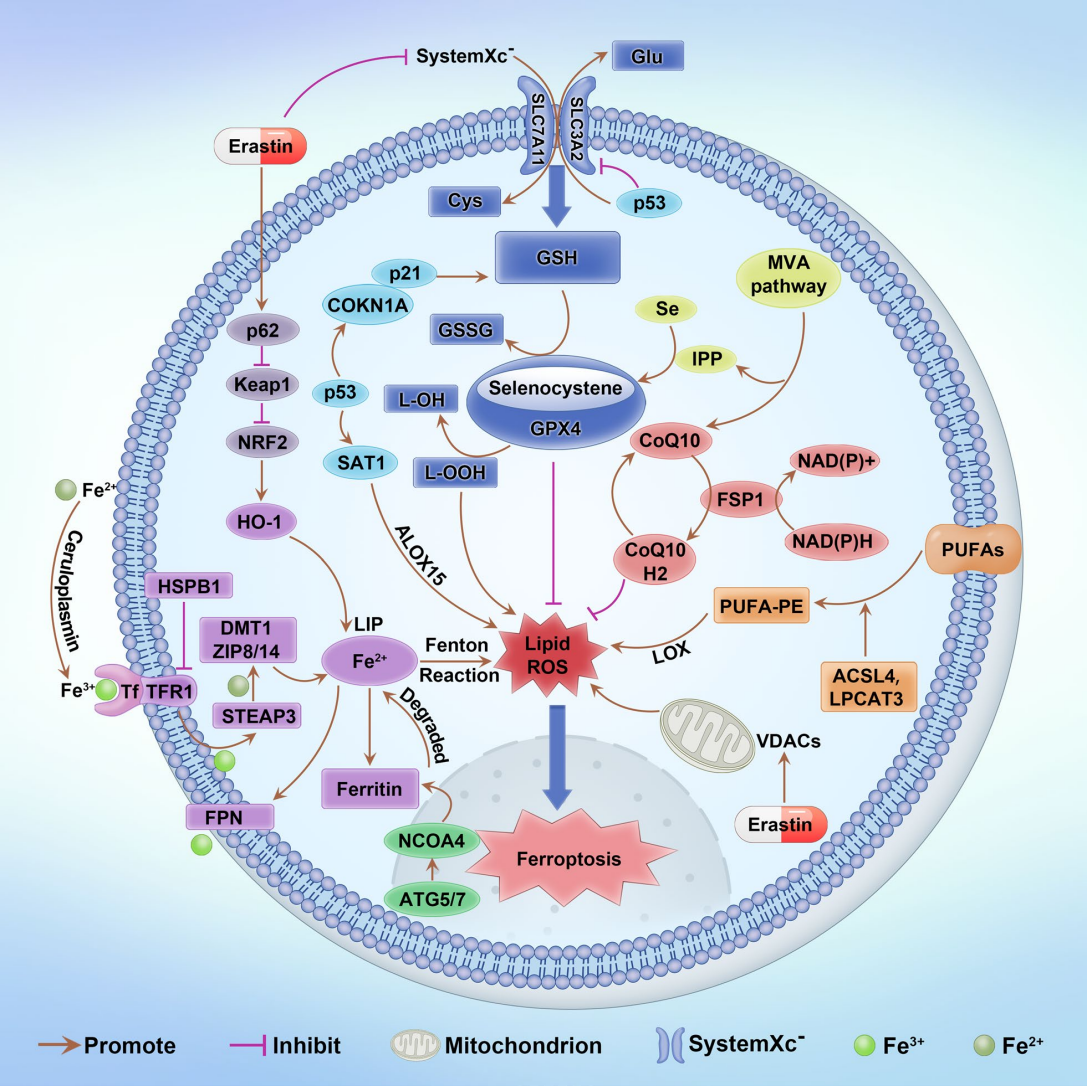
The main regulatory pathways of ferroptosis. The first pathway is regulated by the inhibition of the system Xc-, MVA pathway and p53 regulatory axis through the GSH/GPX4 pathway. The second pathway is regulated by Keap1/HO-1, the ATG5-ATG7/NCOA4 pathway and STEAP3. The next pathway is the regulation of lipid metabolism through P53/ALOX15, ACSL4 and LPCAT3. Finally, the NAD(P)H/FSP1/CoQ10 pathway regulates iron-mediated death in concert with GPX4

Iron metabolism plays an important role in the occurrence and development of ferroptosis. In the absence of disease, iron metabolism operates normally, and the transfer of iron into and out of the cell remains stable. In contrast, excessive accumulation of iron can cause damage to an organism [39]. However, it remains unclear whether iron levels determine the development of ferroptosis in response to disease. What is certain is that sustained increases in iron intake and decreases in iron efflux stimulate oxidative damage, thereby leading to ferroptosis (Fig. 1).

### Lipid peroxidation associated with ferroptosis in vivo

Lipid peroxidation is not only an important marker of ferroptosis but also a cause of ferroptosis. Free polyunsaturated fatty acids (PUFAs) are important substrates for lipid oxidation, and PUFAs in cell membranes are important targets for reactive oxygen species (ROS) attack [40]. Lipid peroxidation occurs due to the reaction between ROS and macromolecules such as polyunsaturated acids and phosphatidylethanolamine (PE). This process also generates lipid peroxidation (LPO), which further generates malondialdehyde (MDA), lipid peroxide (LOOH) and 4-hydroxynonenal (4-HNE) [41]. The free radicals generated by these LPOs can damage biological membranes and affect the function and structure of cells. In addition, adrenal acyl (AdA) is synthesized into free fatty acids (FFAs) via acyl coenzyme A synthase long chain family member 4 (ACSL4) and arachidonic acid (AA). In the final step, lipoyl coenzyme B, which is esterified by lysophosphatidylcholine acyltransferase 3 (LPCAT3), interacts with PE to produce PUFA-PE [42]. PUFA-PE is further lipid peroxidized by lipoxygenase (LOX) and releases ROS and phospholipid hydroperoxides [43]. Therefore, as the ROS concentration continues to increase beyond the normal physiological range, it will further affect biofilm function and structure, causing ferroptosis [44]. In summary, we suggest that interfering with ferroptosis by regulating ACSL4, LPCAT3 and LOX may be a new strategy to combat disease (Fig. 1).

### Regulation of Ferroptosis

#### The System Xc-/GSH/GPX4 Axis

System Xc-, which consists of solute carrier family 7 member 11 (SLC7A11) and solute carrier family 3 member 2 (SLC3A2), is distributed in phospholipid bilayers and is one of the antioxidant systems in cells. l-Glutathione (GSH), an important antioxidant in the oxidative stress response, is composed of glycine, glutamate and cysteine and is present as reduced GSH and oxidized glutathione (GSSG) [45]. Selective inhibition of System Xc- decreases intracellular GSH levels, increasing the accumulation of ROS and ultimately inducing ferroptosis [46]. P53, activating transcription factor 3 (ATF3), and BRCA1-associated protein 1 (BAP1) enhance ferroptosis by significantly reducing the expression level of SLC7A11 [47, d]. Glutathione peroxidase 4 (GPX4), an important characteristic marker of ferroptosis, is a GSH-dependent antioxidant. GPX4 promotes the reduction of phospholipid hydroperoxides (PLOOH) in cells and can inhibit ferroptosis in cells by converting PLOOH to nontoxic lipid alcohols [49, 50].

#### The p62/Keap1/Nrf2 Axis

p62/SQSTM1 (p62) is an intracellular oxidative stress-induced protein and a receptor for ubiquitinated proteins and organelles [51]. Nuclear factor erythroid 2-related factor 2 (Nrf2) is a key regulator of intracellular oxidative stress [44]. Kelch-like ECH-associated protein 1 (Keap1) is rich in cysteine residues, which in turn leads to inactivation of Keap1, which induces the translocation of Nrf2 to the nucleus, which further activates the antioxidant protein HO-1 [52, 53]. Moreover, a continuous increase in Nrf2 nuclear translocation can upregulate the protein expression of the downstream factor HO-1, which can alleviate ferroptosis [54]. Therefore, disease control can be achieved by alleviating ferroptosis through the p62/Keap1/Nrf2 pathway [55].

#### The FSP1/NAD(P)H/CoQ10 Axis

Ferroptosis suppressor protein 1 (FSP1) has been suggested to be a survival factor [56]. Coenzyme Q10 (CoQ10) is a fat-soluble quinone compound and is present in the oxidized ubiquinone form (CoQ), the semioxidized semiquinone form (CoQH) and the fully reduced ubiquinoline form (CoQH2) [57]. NAD(P)H is a typical coenzyme that can play a role in the anabolic pathway [58]. FSP1 can promote the regeneration of COQ10 through NAD(P)H. FSP1/NAD(P)H/CoQ10 and GPX4/GSH synergize with each other to inhibit ferroptosis [59].

#### The P53/SAT1/ALOX15 Axis

P53, which is a factor that mediates the cell cycle, cellular senescence and apoptosis, has recently been shown to promote ferroptosis [60]. SAT1 is not only a restriction enzyme for polyamine catabolism but also a transcriptional target gene of P53. It has been shown that P53 upregulates the expression level of arachidonic acid lipoxygenase 15 (ALOX-15) by activating SAT1, which in turn leads to lipid peroxidation and ferroptosis induced by the accumulation of ROS [61]. For example, in a study by Yang, Ma, Li, Ling, Zhou, Chu, Xue and Tao [62], inhibiting ferroptosis and mitigating acute lung injury could be achieved by regulating the expression of P53. However, P53 may have bidirectional effects on the regulation of ferroptosis, and the exact mechanism needs further study.

#### Other axes

Mevalonate (MVA) is another pathway that regulates ferroptosis. IPP and COQ10 are important products of the MVA pathway, and IPP regulates selenocysteine tRNA to enhance GPX4 expression, thereby regulating the development of iron prolapse [63]. The GCH1/DHFR/BH4 [25, 64] and ATG5/ATG7/NCOA433 [65] pathways also play roles in regulating ferroptosis by regulating intracellular iron ion and ROS formation (Fig. 1).

## The relationship between osteoporosis and ferroptosis

### Ferroptosis and osteoblasts

**Fig. 2:**
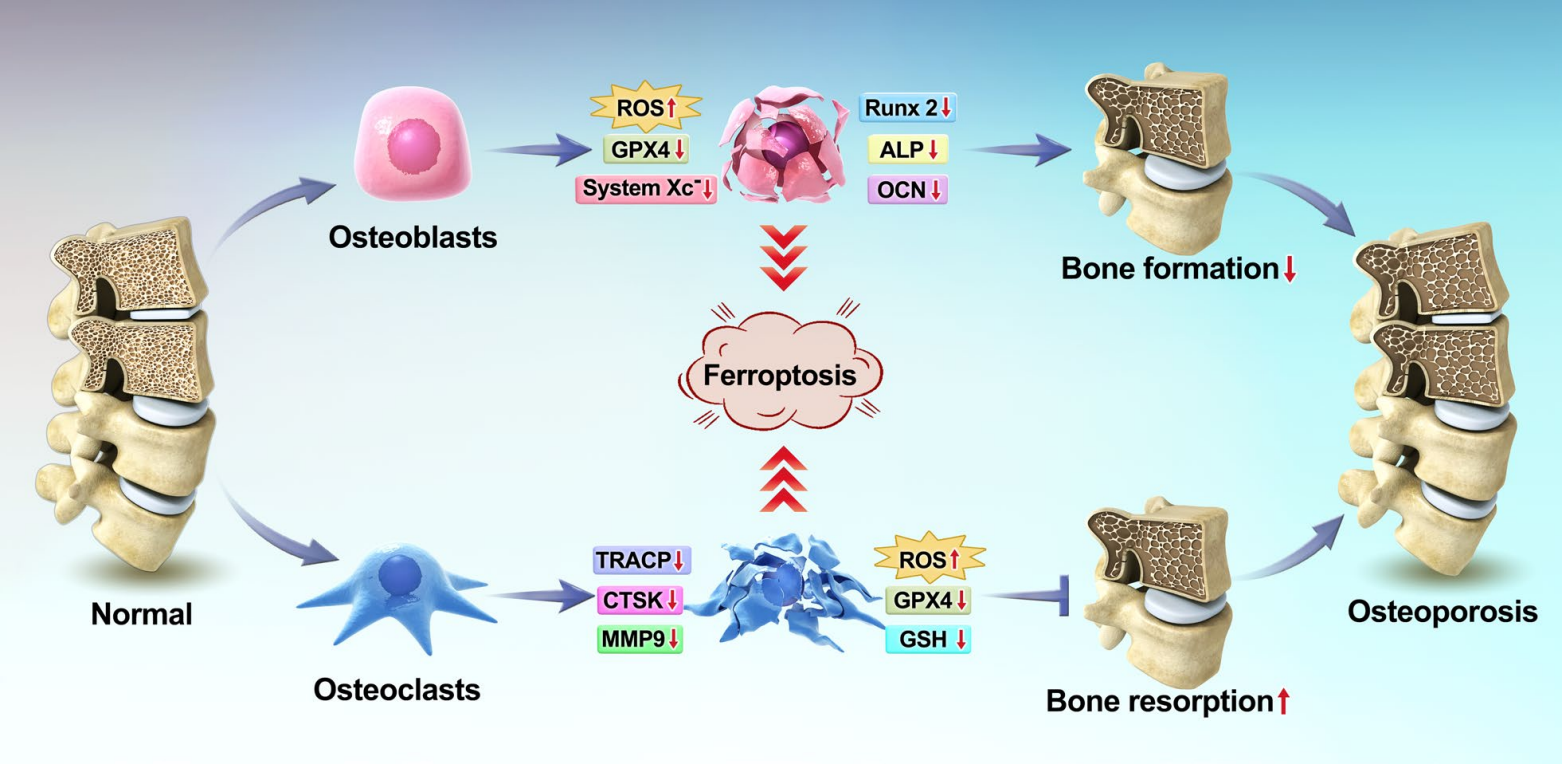
Relationship between ferroptosis and osteoporosis. First, OBs undergo ferroptosis, resulting in decreased osteogenic capacity and decreased bone formation. Second, OCs do not undergo ferroptosis, resulting in increased osteoclastic capacity and increased bone resorption

OBs are responsible for bone formation, and osteoporosis can be prevented and treated by promoting the proliferation of OBs [66]. Iron accumulation causes an excess of ROS, which induces bone metabolic signaling pathways that further inhibit OB activity and inhibit bone resorption [67, 68]. Previous studies have shown that ferroptosis inhibits the abilities of MC3T3 cells [69] and bone marrow mesenchymal stem cells (BMSCs) [70] to undergo osteogenic differentiation, affecting the onset and progression of osteoporosis. This may be due to the overexpression of DMT1 in OBs, which causes oxidative stress and inhibits the osteogenic function of OBs [71]. A significant increase in ROS and a significant decrease in GPX4 were observed in an in vitro model of high glucose-induced MC3T3 cells, and cells with smaller mitochondria and membranes with darker staining and obvious membrane folding were observed, suggesting that MC3T3 cells that underwent ferroptosis had significantly reduced differentiation toward OBs and formed mineralized nodules [28, 72]. Mitochondrial ferritin (FtMt) maintains intracellular apposition homeostasis by reducing the amount of free Fe^2+^ in mitochondria, decreasing ROS levels, and reducing oxidative stress [73]. It was confirmed that increased expression of mitochondrial DMT1 in OBs led to iron overload in a high glucose environment and that the overexpression of FtMt reduced intracellular ROS levels and inhibited ferroptosis in OBs [72]. Therefore, inhibiting ferroptosis in OBs may be a therapeutic strategy to combat osteoporosis (Fig. 2).

### Ferroptosis and osteoclasts

OCs, which are responsible for bone resorption, are multinucleated giant cells formed by the fusion of mononuclear macrophage precursor cells induced by macrophage colony-stimulating factor (M-CSF) and receptor activator of nuclear factor-κB ligand (RANKL) [74]. The expression of the prostaglandin endoperoxide synthase 2 gene, changes in the levels of malondialdehyde, reduced GSH and Fe2 + levels, and iron deposition in mitochondria occurred in bone marrow-derived macrophages (BMDMs) induced by RANKL stimulation [75]. In addition, iron ions can activate the MAPK and NF-κB pathways through the continuous accumulation of ROS, increasing the differentiation of OCs and promoting bone loss [76]. The iron chelator DFO reduces the iron levels in cells and inhibits the proliferation and differentiation of OBs by inhibiting the MAPK signaling pathway and affecting the expression levels of downstream NFATc1, C-FOS and C-Myc [77]. Another study showed that zoledronic acid could induce ferroptosis in OCs by promoting the ubiquitination and degradation of p53 [78]. Therefore, promoting ferroptosis in OCs may be an additional therapeutic strategy to combat osteoporosis (Fig. 2).

### Ferroptosis, a new therapeutic target in natural products for the prevention and treatment of osteoporosis

China is one of the most experienced countries in the world in using natural products to treat diseases. In ancient China, doctors have already used natural products to treat osteoporosis, such as *Epimedium, Scutellaria baicalensis*, *Eucommia ulmoides* etc. Since the concept of " ferroptosis " was proposed in 2012, an increasing number of natural products have been proven to have anti osteoporosis effects by regulating ferroptosis. As we know, the essence of osteoporosis is an imbalance between osteoblasts and osteoclasts. It should be noted that the mechanism of regulating osteoblasts and osteoclasts is generally mediated by natural products that interfere with the ferroptosis regulatory pathway, such as System Xc -/GSH/GPX4 axis, p62/Keap1/Nrf2 axis, and FSP1/NAD (P) H/CoQ10 axis mentioned above. The following is an overview of how some natural products exert anti osteoporosis effects by regulating ferroptosis.

Artemisinin (ARS) is the main extract of the Chinese herb *Artemisia annua*, which has antimalarial [79] and anticancer effects [80] and has recently been shown to inhibit OCs. Previous studies have shown that ARS can inhibit bone loss in animal models, including lipopolysaccharide (LPS) induced bone loss models [81], ovariectomized osteoporosis models [82], titanium particle induced osteolysis models [83], and osteoarthritis induced bone loss models [84]. According to the high level of iron in osteoclasts, ARS may inhibit osteoclast differentiation through mechanisms related to intracellular iron. This mechanism involves mediating P53/SAT1/ALOX15 axis to block intracellular oxidative damage, peroxides, and increase cellular free iron levels to induce ferroptosis in osteoclasts [85]. The activation of ARS by iron and the high iron content in osteoclasts may activate the ARS peroxide group to produce a large number of free radicals, thereby inhibiting the generation and bone resorption of osteoclasts [86]. In addition, it can also cause ferroptosis in OCs by downregulating the RANKL-induced osteoclastogenesis pathway [86, 87].

Gastrodin is a major component of the Chinese herbal medicine asparagine, which modulates neurotransmitters [88] and has anti-inflammatory [89] and antioxidant effects [90]. Currently, asparagine is widely used in the prevention and treatment of osteoporosis [92, 94, b]. Both in vivo and in vitro studies have confirmed that gastrodin reduces glucocorticoid induced cell apoptosis and increases mitochondrial membrane function by activating the NRF2/HO-1 pathway, inhibits ferroptosis of osteoblasts, enhances differentiation function of osteoblasts, and thus achieves the effect of improving osteoporosis [93].

Biochanin A, which is a major component of *Astragalus membranaceus*, has been shown to have osteoprotective effects in vivo and in vitro [94, 95]. The mechanism of action may involve reducing intracellular iron levels by inhibiting TFR1 and promoting FPN expression or by inhibiting ferroptosis by preventing lipid peroxidation through the Nrf2 and System Xc-/GPX4 signaling pathways [95]. Astragalus polysaccharide is another major active ingredient in Astragalus membranaceus. The ferroptosis model of BMSCs induced by ferric ammonium citrate was found after intervention with astragalus polysaccharides. Astragalus polysaccharide can effectively reduce the accumulation of intracellular and mitochondrial ROS in BMSCs by intervening in p62/Keap1/Nrf2 axis, thereby protecting BMSCs from ferroptosis, ultimately restoring cell proliferation and differentiation ability, and increasing bone mass [96].

Quercetin is widely found in TCM, such as *Scutellaria baicalensis* [97], *Ginkgo biloba* [98] and *Eucommia japonica* [99]. Quercetin has been proven to be effective in preventing and treating osteoporosis by inhibiting osteoclasts and promoting osteoblasts [100, 101]. By detecting Fe3 + reduction and lipid peroxidation clearance rates, researchers found that quercetin can significantly reduce ROS accumulation and protect BMSCs from erastin induced ferroptosis, thereby improving osteoporosis [102]. And this mechanism may be achieved through antioxidant pathways, such as the NRF2/HO-1 ferroptosis pathway.

The effective extract of *Curculigo orchioides* is the phenolic glycoside curculigoside, which has shown antioxidant and bone protective properties [103]. The phenolic glycoside curculigoside can protect the proliferation and differentiation ability of MC3T3-E1 induced by excessive iron by upregulating the levels of FoxO1 and Nrf2, downregulating the levels of p53 and FoxO1 phosphorylation, enhancing its antioxidant effect, inhibiting cell ferroptosis, and enhancing the activity of ALP. In addition, it can improve the bone density and microstructure of iron excess mice [104].

Resveratrol, as an activator of SIRT1, extracts dietary foods such as pistachios, peanuts, etc. Studies have shown that resveratrol can significantly protect bone trabecular defects and injuries in iron excess mice, so as to prevent bone loss in osteoporosis mice. The mechanism may be that resveratrol upregulates FoxO1 to protect against excessive iron damage to Runx2, OCN, and type I collagen, reducing oxidative stress and alleviating cell ferroptosis. In addition, resveratrol also reduced the proportion of OPG/RANKL in osteoblasts and mice, and improved bone loss [105].

Icariin is a flavonoid glycoside extracted from *Herba Epimedii*, which can play an antiosteoporosis role [106]. In vitro studies have shown that icariin can reverse Runx2, ALP and OCN by inhibiting ROS production and mitochondrial membrane potential dysfunction caused by iron overload in osteoblasts, thereby protecting osteoblasts from ferroptosis. In addition, icariin can also inhibit osteoclast differentiation and function. Meanwhile, icariin can significantly reduce the production and accumulation of iron in the bone marrow, promote osteoclast ferroptosis, and thus inhibit bone loss in animal models [107].

Neferine is a natural product extracted from *Nelumbo nucifera* and has significant anti-inflammatory [108], antioxidant [109], and anticancer properties [110]. Neferine exerts therapeutic effects by regulating the Nrf2/HO-1 pathway to control cell ferroptosis [111]. Similarly, Neferine can use NF-κB signaling pathway inhibits osteoclasts and promotes the generation, proliferation, and differentiation of osteoblasts, preventing and treating osteoporosis [112].

Curcumin is the main active ingredient of traditional Chinese medicine *Curcuma longa LINN*, belonging to the polyphenolic yellow substance [113]. A study in vitro showed that Curcumin upregulated the phosphorylation level of AKT/GSK3β, improved mitochondrial oxidation status, inhibited the death of the osteoblast line Saos-2, and promoted its osteogenic function [114]. Another in vivo study also confirmed that curcumin exerts an antiosteoporosis effect by protecting osteoblasts from death [115].

Artesunate is one of the artemisinin compounds derived from the plant Artemisia annua [116]. Artesunate can induce ferroptosis in osteoclasts by increasing the production of malondialdehyde and 4-hydroxynonanal. This study also confirms that Artesunate plays a role in inhibiting the proliferation and differentiation of osteoclasts, reducing bone loss [117].

Maresin1 is a major derivative of -3 fatty acids, which has been proven to have antioxidant and anti-inflammatory effects [118]. A recent experimental result indicates that Maresin1 primarily activates the NRF2 signaling pathway, further increasing the activity of GPX4 and SLC7A11, achieving inhibition of ferroptosis in osteoblasts and promotion of osteogenic ability in MC3T3-E1 cells. Maresin1 inhibits type 2 diabetes osteoporosis based on this mechanism [119].

Silymarin is a flavonoid compound extracted from milk thistle seeds with significant antioxidant properties [120]. Silymarin has been confirmed to enhance the expression of RUNX2 and SIRT1, inhibit ferroptosis in osteoblasts, and thus promote the activity and differentiation of osteoblasts. At the same time, it was found in animal models of osteoporosis that Silymarin can improve bone loss by inhibiting ferroptosis [121].

Humulus lupulus L is a traditional folk medicine in China that can be used for postmenopausal osteoporosis [122]. Xanthohumol is a unique hop extract with anti-inflammatory, antioxidant, and osteoprotective effects [123–125]. Xanthohumol can be activated by the AKT/GSK3β/ Nrf2 pathway, inhibits oxidative stress induced by iron dextran, inhibits ferroptosis in osteoblasts, and effectively improves bone loss and increases bone microstructure in mice with iron overload. In addition, Xanthohumol significantly promoted the cell proliferation and differentiation ability of osteogenic cells induced by iron dextran, and the expression of osteogenic-related proteins such as Runx2, thereby enhancing the expression of ALP [126].

Geniposide is an effective extract from gardenia flowers and plays an important role in combating osteoporosis [127]. In vitro and in vivo studies have confirmed that Geniposide exerts antioxidant stress by directly upregulating the RNA binding protein Grsf1 of GPX4, inhibiting cell ferroptosis [128], and regulating NRF2/NF-κB signaling pathway inhibits osteoblast death and exerts an antiosteoporosis effect [129].

Herbal compounding is also widely used in the prevention and treatment of osteoporosis through the ferroptosis pathway. Qing'e Pill is an herbal formula consisting of four botanicals with strong antioxidant activity against lipid metabolism dysfunction [130]. In vitro studies have shown that Qingmoth Pill can inhibit ferroptosis by affecting the System Xc-/GPX4 signaling pathway, thereby promoting the differentiation function of OBs. It was also confirmed that Qingmoth Pill improved erastin-induced ferroptosis in depressed rats in vivo [131]. These in vivo and in vitro studies have confirmed that herbs and herbal compounds can prevent and treat osteoporosis through ferroptosis (Table 1).

**Table 1:** Examples of natural products for the prevention and treatment of osteoporosis through ferroptosis

Natural products	Mechanisms related to ferroptosis regulation	Mechanism of the prevention and treatment of osteoporosis	References
Artemisinin	Increased TFR1-mediated iron uptake	Promotion of OC differentiation	[86, 87]
Gastrodin	Activation of the NRF2/HO-1 pathway	Enhanced differentiation of OB to improve OB function	[91, 93]
Biochanin A	Alters the Nrf2 and System Xc-/GPX4 signaling pathways to prevent lipid peroxidation	Inhibition of OC differentiation	[95]
Astragalus polysaccharide	Reduce the accumulation of ROS in mitochondria	Promoting the proliferation and differentiation of OB	[96]
Quercetin	The antioxidant pathway reduces ROS accumulation	Protects OB from damage	[102]
Phenolic glycoside curculigoside	Upregulation of FoxO1 and Nrf2 levels, downregulation of p53 and FoxO1 phosphorylation levels	Enhance the proliferation and differentiation ability of osteoblasts	[104]
Resveratrol	protects Runx2, OCN and type I collagen	Inhibition of osteoclasts	[105]
Icariin	Inhibition of ROS production and mitochondrial membrane potential dysfunction	Inhibition of osteoclasts and protection of osteoblasts	[107]
Neferine	By regulating the Nrf2/HO-1 pathway to control cell ferroptosis	Inhibition of osteoclasts and protection of osteoblasts	[111, 112]
Curcumin	Regulating AKT/GSK3βpathway to improve mitochondrial oxidative status	Inhibited the death of the osteoblast line Saos-2	[114, 115]
Artesunate	Increasing the production of malondialdehyde and 4-hydroxynonanal	Inhibiting the activity and differentiation of osteoclast	[117]
Maresin1	Activating the NRF2 signaling pathway, further increasing the activity of GPX4 and SLC7A11	Inhibiting the ferroptosis in osteoblasts and promoting the osteogenic ability of MC3T3-E1 cells	[119]
Silymarin	Enhancing the expression of RUNX2 and SIRT1	Inhibiting the ferroptosis in osteoblasts	[121]
Xanthohumol	Activating the AKT/GSK3β/ Nrf2 pathway	Protecting osteoblasts from ferroptosis	[126]
Geniposide	Antioxidant via directly upregulating Grsf1 of GPX4	Inhibiting the osteoblast death	[128, 129]
Qing'e Pill	Affects the System Xc-/GPX4 signaling pathway	Promotes the differentiation of OB	[131]

## Discussion

Osteoporosis has been effectively controlled, but the commonly used anti-osteoporosis drugs in clinical practice have shortcomings, such as unstable efficacy, serious toxic side effects, and susceptibility to drug resistance [132]. In recent years, with the continuous research on natural products in East Asian countries, it has been found that compared with traditional synthetic drugs, natural products have a larger molecular weight, stable active skeleton, and excellent biological activity in the process of anti- osteoporosis [133–135]. Geniposide has obvious advantages in the treatment of osteoporosis, such as high biological activity and multiple therapeutic mechanisms [127, 129]. It is precisely based on these advantages of natural products that it has become the most common choice for the development of new drugs against osteoporosis [136]. Both clinical and basic experiments have shown that natural products have enormous to exert anti-osteoporosis effects. Ferroptosis is another form of cell death distinct from autophagy and apoptosis. Interestingly, ferroptosis has also been widely used in the treatment of osteoporosis. This mechanism mainly inhibits osteoblasts' ferroptosis and promotes osteoclasts' ferroptosis, thereby reducing bone loss and achieving anti-osteoporosis effects. Multiple in vivo and in vitro studies have confirmed this conclusion [137–139]. Various natural products, such as Gastrodin, Biochanin A, and Icariin, have been proven to have anti-osteoporosis effects through ferroptosis. Combining natural products and cell ferroptosis is another practical therapeutic approach for preventing and treating osteoporosis.

Although many natural products have been proven to have anti-osteoporosis effects by regulating ferroptosis, this evidence is limited to the cellular or animal level, and there are still very few clinical drugs for treating osteoporosis through the conversion of natural products, which is a significant limitation and challenge. On the one hand, screening and verifying effective drugs that can effectively treat osteoporosis in clinical practice from natural products that have been proven to have effects in both cells and animals requires a considerable workload, human resources, and economic expenses, as well as a significant amount of time, which is very detrimental to the conversion into clinical drugs [140]. Therefore, this requires more efficient and cutting-edge technology development and precise identification, which may be a good suggestion to address this reason. On the other hand, natural products have drawbacks such as fast metabolism, poor absorption, low bioavailability, and low specificity [141]. One way to address this drawback is for researchers to focus on improving the bioavailability and specificity of natural products by developing new drug delivery systems. In addition, the limitations of natural product collection and safety are also why it is difficult to convert into clinical drugs [142]. The reasons listed above require our researchers to continuously explore safer, more efficient, precise, and more suitable natural products for clinical conversion.

## Conclusion

In this review, we summarized iron metabolism, lipid peroxidation and the pathways associated with ferroptosis in vivo. We also detailed how ferroptosis regulates OBs and OCs to prevent and treat osteoporosis. Besides, some in vivo and in vitro examples of natural products for preventing and treating osteoporosis through ferroptosis were discussed. Finally, we discussed the advantages and disadvantages of natural products and the effective way for natural products to exert anti-osteoporosis effects by mediating ferroptosis. In conclusion, this review provides a theoretical basis for studying the mechanism of ferroptosis and the relationship between ferroptosis and osteoporosis to guide natural products in the prevention and treatment of osteoporosis. Furthermore, natural products have great potential to regulate OBs and OCs by mediating ferroptosis to prevent and treat osteoporosis, and it is worthwhile to explore and discover more natural products that can prevent and treat osteoporosis.

## Abbreviations

OCs: Osteoclasts
OBs: Osteoblasts
Runx2: Runt-related transcription factor 2
OCN: Osteocalcin
OPN: Osteopontin
BSP: Bone salivary protein
TCM: Traditional Chinese medicine
TFR1: Transferrin receptor 1
DMT1: Divalent metal transporter protein 1
PUFAs: Polyunsaturated fatty acids
ROS: Reactive oxygen species
PE: Phosphatidylethanolamine
LPO: Lipid peroxidation
LOOH: Lipid peroxide
ACSL4: A synthase long chain family member 4
LOX: Lipoxygenase
SLC7A11: Solute carrier family 7 member 11
SLC3A2: Solute carrier family 3 member 2
GSH: L-Glutathione
GSSG: Oxidized glutathione
GPX4: Glutathione peroxidase 4
PLOOH: Phospholipid hydroperoxides
Nrf2: Nuclear factor erythroid 2-related factor 2
Keap1: Kelch-like ECH-associated protein 1
FSP1: Ferroptosis suppressor protein 1
CoQ10: Coenzyme Q10
MVA: Mevalonate
BMSCs: Bone marrow mesenchymal stem cells
M-CSF: Macrophage colony-stimulating factor
RANKL: Receptor activator of nuclear factor-κB ligand
BMDMs: Bone marrow-derived macrophages
ARS: Artemisinin
